# Sleep quality, personal and work variables and life habits of hospital nurses

**DOI:** 10.1590/1518-8345.5756.3538

**Published:** 2022-05-16

**Authors:** Andressa Fernanda Silva, Rita de Cássia de Marchi Barcellos Dalri, Alan Luiz Eckeli, António Neves Pires de Sousa Uva, Aida Maria de Oliveira Cruz Mendes, Maria Lúcia do Carmo Cruz Robazzi

**Affiliations:** 1 Universidade de São Paulo, Escola de Enfermagem de Ribeirão Preto, Centro Colaborador da OPAS/OMS para o Desenvolvimento da Pesquisa em Enfermagem, Ribeirão Preto, SP, Brasil.; 2 Bolsista da Coordenação de Aperfeiçoamento de Pessoal de Nível Superior (CAPES), Brasil.; 3 Universidade de São Paulo, Faculdade de Medicina de Ribeirão Preto, Ribeirão Preto, SP, Brasil.; 4 Universidade de Nova Lisboa, Escola Nacional de Saúde Pública, Lisboa, Portugal.; 5 Escola Superior de Enfermagem de Coimbra, Unidade de investigação em Ciências da Saúde: Enfermagem, Coimbra, Portugal.; 6 Universidade Federal da Paraíba, João Pessoa, PB, Brasil.

**Keywords:** Occupational Health, Nursing, Shift Work, Sleep, Sleep Disorders, Hospitals, Saúde do Trabalhador, Trabalho em Turnos, Enfermagem, Sono, Transtornos do Sono, Hospitais, Salud Laboral, Horario de Trabalho por Turnos, Sueño, Trastornos del Sueño-Vigilia, Hospitales

## Abstract

**Objective:**

to identify the possible associations between sleep quality, personal and work variables and the life habits of hospital nurses.

**Method:**

a cross-sectional, exploratory, correlational and quantitative study, carried out from October to December 2019. The data were collected with the application of a questionnaire that addressed the respondents’ personal characteristics, life habits and working conditions. The *Pittsburgh Sleep Quality Index (PSQI)*, Brazilian Portuguese version, was used to assess sleep quality.

**Results:**

the participants were 42 professionals: 31 (73.8%) women, aged between 26 and 66 years old (mean of 40.2); 61.9% worked overtime; 26.2% had two employment contracts and 40.5% had absences from work. Sleep quality was considered good by 9.5% of the participants, poor by 64.3% and categorized as with sleep disorders by 26.2%. In the population that worked rotating shifts, this quality was identified as poor by 26.2%. The worst results were found in the age group from 30 to 39 years old and there was a statistical significance in the “living with a partner” variable.

**Conclusion:**

there was impairment in the nurses’ sleep quality and there is a need to monitor these workers, particularly those who work in shifts, in order to provide preventive measures to mitigate the harms to their health.

## Resumo

**Objetivo:**

identificar as possíveis associações entre a qualidade do sono, as variáveis pessoais e laborais e os hábitos de vida de enfermeiros hospitalares.

**Método:**

estudo transversal, exploratório, correlacional, quantitativo, realizado no período de outubro a dezembro de 2019. Os dados foram coletados com a aplicação de um questionário que abordou as características pessoais, hábitos de vida e as condições de trabalho dos pesquisados. Para avaliação da qualidade do sono, utilizou-se a *Pittsburgh Sleep Quality Index (PSQI)*, versão do português do Brasil.

**Resultados:**

participaram 42 profissionais, 31 (73,8%) mulheres, entre 26-66 anos (média de 40,2); 61,9% realizavam horas extras; 26,2% possuíam duplo vínculo empregatício e 40,5% tiveram ausências no trabalho. A qualidade do sono foi considerada boa por 9,5% dos participantes, má por 64,3% e com distúrbios do sono por 26,2%. Na população que realizava turnos rotativos, essa qualidade foi identificada como má por 26,2%. Os piores resultados foram encontrados na faixa etária de 30-39 anos e houve significância estatística na variável “viver com companheiro(a)”.

**Conclusão:**

houve prejuízo na qualidade de sono dos enfermeiros; há a necessidade de monitoramento desses trabalhadores, particularmente dos que realizam trabalhos em turnos, com o intuito de propiciar medidas preventivas, visando mitigar os danos à sua saúde.

## Resumen

**Objetivo:**

identificar posibles asociaciones entre la calidad de sueño, las variables personales y laborales y los estilo de vida de los enfermeros de hospital.

**Método:**

estudio transversal, exploratorio, correlacional, cuantitativo, realizado de octubre a diciembre de 2019. Los datos fueron recolectados mediante un cuestionario que abordó las características personales, estilo de vida y condiciones de trabajo de los encuestados. Para evaluar la calidad de sueño, se utilizó el *Pittsburgh Sleep Quality Index (PCSI)*, versión en portugués de Brasil.

**Resultados:**

participaron 42 profesionales, 31 (73,8%) mujeres, con edad entre 26 y 66 años (media 40,2); el 61,9% trabajaba horas extras; el 26,2% tenía dos vínculos laborales y el 40,5% faltó al trabajo. La calidad de sueño fue considerada buena por el 9,5% de los participantes, mala por el 64,3% y con trastornos del sueño por el 26,2%. El 26,2% de la población que hacía turnos rotativos, calificó la calidad como mala. Los peores resultados se encontraron en la franja etaria de 30 a 39 años y hubo significación estadística en la variable “vive en pareja”.

**Conclusión:**

la calidad de sueño de los enfermeros se vio afectada; es necesario monitorear a estos trabajadores, en particular a los que trabajan por turnos, a fin de implementar medidas preventivas que reduzcan los daños a su salud.

**Highlights**

(1) Sleep Quality (SQ) in hospital nurses who work in shifts has been impaired.

(2) Poor SQ can result in illness, decreased productivity and work-related accidents.

(3) There is a need to monitor nurses who work in shifts.

(4) Preventive measures can mitigate the harms to the health of these workers.

## Introduction

Sleep is necessary for life and corresponds to a stage for repairing the body’s physiological activities^1^. Enjoying good Sleep Quality (SQ) is important for health^2^ because some toxin clearance mechanisms occur during this period, which is crucial for immune^3^, cardiovascular^4^, reproductive^5^ and endocrine^6^ functions and for pain control^7^. Sleep acts on the cognitive functions, memory consolidation and information storage, being essential for the balance of the human body^7^. SQ and its duration can be considered important Quality of Life (QoL) indicators^8^, meaning that sleep must be restorative, with adequate duration, depth and quality, favoring the person to wake up in a good mood^9^.

Qualitatively inadequate sleep periods compromise the body homeostasis mechanisms. Extrinsic factors can influence SQ, such as: environment, climate, exposure to light and electronic devices before bedtime^10^, work activities^2^, eating habits, absence of a routine in relation to the times to go to sleep to wake up, and consumption of alcoholic beverages, other drugs and medications^11^. Intrinsic factors also exert an influence, such as health status and sedentary lifestyle^12^. SQ can also be influenced by work^2^, and shift work can exert negative impacts on it, as it favors irregularities in the circadian rhythms, imposed by shift alternation^13^.

In hospitals, the health professionals’ work is uninterrupted, with alternating shifts and with occasional differences in the working hours^13^. Nursing workers correspond to a group with irregular sleep parameters^14^; in some situations, they are subjected to shifts in a fixed or alternating way and the consequences for their health are usually harmful^15^. 

In France, shift work involves 20% of the workforce and is associated with deleterious cardiovascular effects, underlined by dyslipidemia^16^; Chinese and North American researchers identified an association between shift work and obesity among nurses and showed that this type of work can play a significant role in the development of this obesity, especially in America, Europe and Australia, and mainly among those who work night shifts^15^.

In the United States of America (USA) there was an association between shift work-related sleep disorder and erectile dysfunction in workers and it was identified that the change in the circadian rhythm can significantly affect erectile function, proving to be a potential risk factor for this dysfunction^17^. In Seoul, Korea, a study indicated that nurses should monitor their sleep duration and develop their own regular sleep schedules to suit their working hours; consequently, hospitals should establish “healthy” schedules to ensure sufficient sleep hours before these professionals’ work schedules^18^. A study conducted with hospital workers in the USA showed that the night shift was a significant predictor of greater chronic fatigue and less satisfaction with the time available for daily tasks and family and social life, while working in 12-hour shifts provided greater satisfaction with daily tasks and periodic life activities; the driving behavior was changed in response to drowsiness and almost one fifth of the participants suffered a car accident or near miss due to inattention or falling asleep while driving^19^.

Changes in health status caused by night work have been discussed in the literature. A North American study measured sleep patterns and predicted cognitive decline in nurses working day and night shifts; sleep was assessed using wrist actigraphs; 90 of these workers from two hospitals participated in the research, 48 from the night shift and 42 from the day shift, in 12-hour shifts; comparisons were made and night work proved to be a disturbing factor for SQ^20^. In Denmark, a cohort study conducted with nurses showed that night work was associated with an increased risk for severe psychiatric disorders^21^. In South Korea, a research study assessed the QoL of 225,541 individuals using the Pittsburgh Sleep Quality Index (PSQI); poor SQ was associated with worse QoL and was more frequent in subjects with anxiety and depression disorders^22^. In a study conducted in Brazil with 104 Nursing professionals from different work shifts, evaluating SQ and QoL, daytime professionals presented better results when compared to their counterparts who worked at night. Those who presented better SQ also had better QoL, showing the importance of synchronizing the biological rhythms across these variables^23^. Another Brazilian study investigated the QoL of 264 Nursing professionals according to their work schedules. Night work was associated with a severe worsening of at least one QoL component; female gender was associated with sleep disorders; and QoL and SQ were closely correlated, that is, the characteristics of the Nursing profession affected these two variables, which were associated^24^.

Among the consequences of night work, it is possible to observe cognitive decline, fatigue and drowsiness^19^ ^-^ ^20^, factors that favor the occurrence of errors and work-related accidents (WRAs)^20^; in addition to that, mental and physical illness can be favored^23^. Epidemiological links are also established between chronic sleep deprivation and chronic non-communicable diseases, which is worrying, given the high power of disability, expense generation for the health systems, morbidity and, finally, the mortality that such diseases can impose on the population^25^.

Although a number of publications have already been related to the work of nurses in several shifts and there is diverse scientific evidence of the problems related to sleep disorders among them^15^ ^-^ ^22^, the current work reality, which still persists, does not seem to change, both nationally and internationally, which motivated this study.

Given the above about the importance of good quality sleep and the reality of shift work performed by some workers, including Nursing professionals, the following guiding question was elaborated for this study: Among Nursing professionals who work in hospitals, which are the associations between SQ and personal, work and lifestyle variables? 

It is intended that the results obtained provide diverse knowledge that can contribute and assist in the mitigation/elimination of the harmful effects of shift work in hospital Nursing workers, thus showing that they require greater attention to avoid illnesses. It is also hoped to collaborate with the studies already carried out on the health of these professionals, demonstrating that investigating sleep and the repercussions of this physiological process becomes necessary in the search for better living and working conditions. 

The objective proposed was to identify the possible associations between sleep quality, personal and work variables and the life habits of hospital nurses.

## Method

The recommendations of the *Revised Standards for Quality Improvement Reporting Excellence*(SQUIRE 2.0) were used to develop this study. This was a cross-sectional, exploratory and correlational study with a quantitative approach to the data; the data collection locus was a public university hospital in the inland of the state of São Paulo, Brazil. 

The population consisted of nurses who worked in open hospitalization units, in clinical and surgical wards, during the day and night shifts, and with fixed or rotating schedules. It was decided to carry out the study exclusively with nurses, because it was during this period that the COVID-19 pandemic outbreak began, causing fears and insecurities among all Nursing team workers, who were beginning to be transferred to the sectors of direct care for patients contaminated by the disease, reducing the number of nursing technicians and nursing, mainly.

The methodological differential of the study is the uniformity of its sample. For the inclusion and exclusion criteria of the participating nurses, the protocol used in the Sleep Laboratory of the Ribeirão Preto Medical School, University of São Paulo, was followed, namely:

- Inclusion: developing care activity; being present on the data collection dates; working in open hospitalization units (ward sectors of the several clinics); filling in the questionnaires delivered; and having worked at least one year in the hospital.
- Exclusion: working in outpatient clinics and/or in closed hospital units (intensive care, surgical center, laboratories, material and sterilization center, post-anesthetic recovery room and hemodialysis, among others); performing administrative tasks and functions (direction of units and Nursing coordination); presenting diagnoses of diseases that can influence the sleep/wake cycle and rhythm, such as endocrine and psychiatric ailments^26^ ^-^ ^28^; having respiratory diseases such as asthma and sleep apnea^29^; using medications for psychiatric treatments^30^; and making use of antiarrhythmics, beta blockers, medications for heart diseases, corticosteroids and medications to control weight^27^ ^-^ ^29^.

The convenience sampling model was adopted to select the participants, and it was decided to choose those who worked in wards/care units due to the similar characteristics of these places in relation to the work demand, as there are differences in the work of hospital nurses according to the sector in which they develop their activities. Thus, it was sought to avoid the occurrence of biases that would compromise the results.

The study population consisted of 75 nurses according to the list disclosed by the institution’s Human Resources (HR) department. Selection of the participants was as follows: once the list of nurses was obtained, 75 were invited to participate in the research; some were on vacation (4) or on leave (4) and on contractual suspension (4); of the remaining individuals (n=63), 8 refused to participate, leaving n=55. By applying the inclusion/exclusion criteria, seven (7) were excluded because they used medications that influenced the sleep/wake cycle, three (3) had not yet worked 1 year and three (3) did not fill in the questionnaires in full; 42 participants completed all stages and comprised the sample.

Data collection was conducted by the first author of this study from October to December 2019, a period during which the COVID-19 pandemic outbreak began, causing fears and insecurities in nurses and in the Nursing teams.

In this hospital, workers are hired in two ways: 1) by the Consolidation of Labor Laws (CLT) regime, with 36 weekly working hours (h) and without authorization to work overtime; or 2) by public tender, under the statutory legal regime, with 30 weekly working hour, with permission to work overtime wage differences between these contract types. The existing work shifts covered the following: fixed day shift (*Turno Diurno Fixo*, TDF) with an 8-hour daily work schedule; fixed night shift (*Turno Noturno Fixo*, TNF) with a 12-hour work schedule and 36 hours of time off, 6-hour rotating day shift (*Turno Diurno Rotativo*, TDR) with double shift adding 12 hours.

As for the evaluation procedures, to characterize the workers, an instrument elaborated by the authors was used, addressing sociodemographic, work and lifestyle issues such as: age; gender; children; presence of a partner; schooling; tobacco use; alcohol and coffee consumption; type of employment contract; work shift; overtime; other job(s); leaves; work-related accidents; double workdays and, if any, work segment in which they were exercised; and time working in their professional function. 

The *Pittsburgh Sleep Quality Index (PSQI)*, in its validated version for Brazilian Portuguese, was used to assess SQ. It consists of 19 self-answered questions and has good internal consistency and factorial validity; it is self-applicable and consists of seven domains, namely: subjective sleep quality, sleep latency, sleep duration, habitual sleep efficiency, sleep disorders, use of sleep medication; and daytime dysfunction. The sum of these items varies from 0 to 21 points and classifies this result into good sleep quality (0-4 points), poor sleep quality (5-10 points) and sleep disorders (11-21 points)^31^ ^-^ ^32^. Values higher than five points indicate poor sleep quality. The study variables and analyses were processed in the *International Business Machines Corporation*(IBM) - SPSS *Statistics*(*Statistical Package of Social Sciences* - SPSS) program^33^, version 25, and the descriptive analyses were performed by means of absolute (n) and relative (%) frequencies. The Shapiro-Wilk normality test was used to evaluate data distribution in relation to the normality deviation, and can be used in small samples^34^. The t test for independent samples was used to evaluate the associations, verifying the differences between the SQ values and the following variables: gender, dependent children and living with a partner, overtime, double employment contract, absences, work-related accidents, tobacco use, caffeine and alcoholic beverages. The t test allows comparing the mean values^34^. ANOVA^34^ was used to assess the associations between age and work shifts. For the statistical analyses, a significance level of p<0.05 and a 95% Confidence Interval (95% CI) were adopted. In relation to the test power; a number of tests (*post-hoc*) were performed and none of them reached 80%^35^.

All the research ethics precepts were respected, with appreciation and approval by the Research Ethics Committee on August 27^th^, 2018, Edict 2,846,414.

## Results 

As for the personal characteristics, the sample consisted of 42 nurses stratified by age: 26-39 years old - 25 participants (59.5%), 40-66 years old - 17 individuals (40.5%) with a mean of 40.2, median of 39.0, standard deviation of 8.9, minimum age of 26 and maximum age of 66 years old. The majority (31%-73.8%) were women, lived with a partner (27%-64.3%) and had dependent children (23%-54.8%); in relation to schooling, 25 (59.5%) had some graduate degree. 

The work-related variables showed that: 31 (73.8%) were under a statutory employment contract; 52.4% worked on a 6-hour TDR with a 12-hour double shift; 7.1% on an 8-hour daily TDF, 33.3% on a 12 by 36-hour TNF, and 7.1% on a 6-hour TDF. As for the working time (in years), the majority (35.7%) had worked for 1-5 years; 21.4%, from 6 to 10 years; 14.3%, from 11 to 15 years and from 16 to 20 years; 7.1%, from 21 to 25 years; 2.4%, from 26 to 30 years; and 4.8%, from 31 to 35 years. With regard to overtime, 61.9% performed it in the last month prior to data collection and the majority (21.4%) worked 60 overtime hours; 71.4% had no other professional activity; and 26.2% of the 12 who did have it, it was in the health area itself. The majority did not have WRAs (88.1%) and, among the 5 who suffered accidents, the causes were cuts with sharps, electric shocks and burns, falls and contact with body fluids/secretions, among others; the majority (59.5%) did not need work leaves. 

Regarding the lifestyle habits, there was predominance of those who did not smoke, with 36 (85.7%); 23 (54.8%) reported that they drank beers, wines and spirits; the consumption frequency was reported once a month, every 15 days, four times a week or on weekly day offs. Coffee intake was high (85.7%) with amounts ranging from one to eight cups/day. Use of medications was usual for 26 (61.9%). 

According to the *Pittsburgh Sleep Quality Index*(PSQI) the SQ assessment proved to be good in 4 participants (9.5%), poor in 27 (64.3%), and with the presence of sleep disorders in 11 (26.2%). As for the global results of PSQI, the values found were as follows: mean of 9.0 and standard deviation of 3.7, with a maximum score of 18 and a minimum of two. These results indicated that only four workers presented good SQ. The results of the mean and standard deviation scores and the PSQI domains are presented below and indicate that the worst results were found in domains 1, 3, 5 and 7 (Table 1).

**Table 1:** Mean values and standard deviation of the scores obtained in the *Pittsburgh Sleep Quality Index* (PSQI) domains among the nurses participating in the study (n=42). State of São Paulo, Brazil, 2019

PSQI domains	Mean (SD)	Median	Minimum	Maximum
1: Subjective sleep quality	1.6 (0.9)	2.0	0	3
2: Sleep latency	1.3 (1.1)	1.0	0	3
3: Sleep duration	1.6 (0.9)	2.0	0	3
4: Habitual sleep efficiency	0.6 (0.9)	0.0	0	3
5: Sleep disorders	1.8 (0.6)	2.0	1	3
6: Use of sleep medication	0.5 (1.0)	0.0	0	3
7: Daytime sleepiness and daytime disturbances	1.6 (0.8)	2.0	0	3

PSQI has seven domains that consist of the 19 questions that are part of the scale. In this study design for the association tests, the overall values of the scale were used. The Shapiro-Wilk Test normality test^34^ of the overall PSQI value was performed, obtaining a value of 0.217.

Table 2 presents the results of the work-related characteristics, along with those of SQ, according to the scores of the *Pittsburgh Sleep Quality Index* in absolute/relative values.

**Table 2:** Work-related characteristics and *Pittsburgh Sleep Quality Index*(PSQI) classifications among the nurses participating in the study (n=42). State of São Paulo, Brazil, 2019

Variables		PSQI value					
		Good (From 0 to 4)		Poor (From 5 to 10)		Sleep disorder (>10)	
		n	%	n	%	n	%
Contract	Statutory	2	6.5	21	67.7	8	25.8
	Working under the Consolidation of Labor Laws	2	18.2	6	54.5	3	27.3
Working hours	Day	1	16.7	2	33.3	3	50.0
	Night	0	0.0	9	64.3	5	35.7
	Rotating	3	13.6	16	72.7	3	13.6
Working time (years)	Up to 5	2	7.1	19	67.9	7	25.0
	>5	2	14.3	8	57.1	4	28.6
Overtime	Yes	2	7.7	17	65.4	7	26.9
	No	2	12.5	10	62.5	4	25.0

Table 3 presents the *Pittsburgh Sleep Quality Index*(PSQI) mean and standard deviation values, according to the participants’ personal characteristics. As for the global values of this scale, age was a characteristic that varied among the population under study; the worst results were observed among those aged 26-39 years old. As for the mean values for SQ, men and those who had a partner presented the worst values and those who had a graduate degree had better SQ. There was statistical significance in the ‘lives with a partner’ variable (p=0.032).

**Table 3:** Mean and standard deviation (SD) values as per the *Pittsburgh Sleep Quality Index* (PSQI) according to the personal characteristics of the nurses participating in the study (n=42). State of São Paulo, Brazil, 2019

		n	Mean (SD)	p-value*	G Power (1-β prob. error)^†^
Age (years old)	26-39	25	9.7 (3.4)	0.140	0.6047006
	40-66	17	7.0 (4.1)		
Gender	Female	31	8.8 (3.8)	0.627	0.0805799
	Male	11	9.5 (3.8)		
Children	Yes	23	9.0 (3.3)	0.971	0.0500000
	No	19	9.0 (4.3)		
Lives with a partner	Yes	27	9.9 (3.6)	0.032	0.6142120
	No	15	7.3 (3.4)		
Schooling	Graduation	17	9.8 (4.0)	0.232	0.6350182
	Graduate education	25	8.4 (3.5)		

*t-test for independent samples; ^†^Calculation of test power

The mean values and standard deviation of the SQ assessment associated with the work-related characteristics are presented below (Table 4). There were differences between the values regarding the work shifts. Those who worked overtime presented the worst SQ results; those with less than five years working in the unit had this quality more compromised; SQ was better among those who had only one employment contract; and the SQ results were worse among those who had to distance from work due to health reasons and those who had already suffered WRAs. These variables did not present statistical significance.

**Table 4:** *Pittsburgh Sleep Quality Index* (PSQI) mean and standard deviation (SD) values according to work-related characteristics among the nurses participating in the study (n=42). State of São Paulo, Brazil, 2019

		n	Mean (SD)	p-value	G Power (1-β prob. error)^‡^
Work shifts	Fixed day shift	6	10.8 (4.2)	0.055^†^	0.5196066
	Fixed night shift	14	10.2 (3.7)		
	Rotating day shift	22	7.7 (3.3)		
Overtime	Yes	26	9.1 (3.9)	0.827*	0.0569377
	No	16	8.8 (3.6)		
Time working in the unit (years)	Up to 5	28	9.5 (3.8)	0.235*	0.2317350
	≥5	14	8.0 (3.5)		
Double employment contract	Yes	12	10.5 (3.5)	0.128*	0.4069019
	No	30	8.5 (3.7)		
Leave from work	Yes	17	10.0 (4.3)	0.216*	0.3368893
	No	25	8.5 (3.3)		
Work-related accidents	Yes	5	10.4 (5.6)	0.382*	0.1080844
	No	37	8.8 (3.5)		

Fixed day shift, 8 h; fixed night shift schedule; 12/36 h rotating day shift 6 h, double 12 h shift, *t-test for independent samples; ^†^ANOVA; ^‡^Calculation of test power

The life habits and SQ results are presented below (Table 5). None of the smokers presented good SQ and, among the non-smokers, only four had good SQ; worse SQ was observed among those who drank alcoholic beverages and used medications, the most used being analgesics (10%-23.8%), contraceptives (10%-23.8%) and anti-inflammatory drugs (6%-14.3%). No statistical significance was found in these variables.

**Table 5:** Life habits and sleep quality classifications according to the global *Pittsburgh Sleep Quality Index* (PSQI) among the nurses participating in the study (n=42), in absolute values and percentages. State of São Paulo, Brazil, 2019

Variables		Good (From 0 to 4)		Poor (From 5 to 10)		Presence of sleep disorder (>10)		Total Group	
		n	%	n	%	n	%	n	%
Smokes	Yes	0	0.0	4	66.7	2	33.3	6	14.3
	No	4	11.1	23	63.9	9	25.0	36	85.7
								42	100.0
Alcoholic beverages	Yes	3	13.0	14	60.9	6	26.1	23	54.8
	No	1	5.3	13	68.4	5	26.3	19	45.2
								42	100.0
Coffee intake	Yes	4	100.0	21	77.8	11	100.0	36	85.7
	No	0	0.0	6	22.2	0	0.0	6	14.3
								42	100.0
Medication use	Yes	2	7.7	17	65.4	7	26.9	26	61.9
	No	2	12.5	10	62.5	4	25.0	16	38.1
								42	100.0

## Discussion

Investigating the nurses’ sleep quality considering variables related to personal and work issues and lifestyle habits corresponds to an important area of studies of the health of these workers, exploring the impact of QoL on the health of the professionals. 

As already mentioned, although the diverse scientific evidence shows the harms caused by altered sleep among Nursing workers^15^ ^-^ ^22^, the current study is added to such evidence, in an attempt to show and reiterate, with greater clarity, the various problems to which they are subjected.

This professional group experiences working hours in shifts, night work and 12-hour workdays. Given the need to provide Nursing care on a continuous basis, these individuals can develop sleep disorders^2^. In this study, SQ and sociodemographic/personal characteristics were assessed of 42 nurses, as well as their work-related characteristics and lifestyle habits, with an age range from 25 to 60 years old or more; however, there was predominance of individuals aged 30-39 years old. 

Most of the participants were women (73.8%), confirming the fact that Nursing remains an essentially female profession^18^. Regarding family constitution, the majority reported the presence of partners and children, results that are similar to those of another study that made this assessment; in this protocol, statistical significance was found in the “living with a partner” variable; the responsibilities inherent to the home and the care of children, which can influence people’s sleep^36^.

It is known that age is one of the factors that alters the sleep patterns and, consequently, its quality. Over the years, and with the physiological changes resulting from age, SQ has been reduced^37^. Age influences the sleep parameters, and the physiological needs change throughout life^38^.

In this study, 73.8% of the interviewees were women and they are more prone to developing sleep disorders due to hormonal factors^39^. However, men presented worse SQ results; they may have night rest impaired by respiratory problems, such as obstructive sleep apnea syndrome and excessive daytime sleepiness; sleep harms can also result from the modern lifestyle, increased pressure at work and psychological stress^40^.

The results showed adults in the productive phase of life and already presenting impaired SQ states, which can lead to health risks. Poor SQ and sleep deprivation favor the emergence of chronic diseases such as Diabetes Mellitus, hypertension and metabolic syndrome, which account for 72% of the causes of death worldwide^41^. Impaired sleep quality and duration are also associated with the occurrence of stroke, cancer, cognitive decline, mental illness and musculoskeletal problems^42^.

The results were worrisome regarding the participants’ SQ, as only 10% met the criteria suggested by the *Pittsburgh Sleep Quality Index*(PSQI), indicative of good SQ. The literature also presents some similar results: there was low SQ among 513 nurses in a research study conducted in China^37^; and, in a survey with 1,253 nurses in five Japanese regions, low SQ was also identified in hospital nurses^43^.

SQ assessment consists of an index that compiles objective (sleep and wake time) and subjective (difficulty sleeping and tiredness) data, investigated by the SQ *Pittsburgh Sleep Quality Index*(PSQI)^44^.

The body’s metabolism, sleep, behavior and disposition at various times of the day and night are regulated by the circadian rhythm, which represents a period of one day (24 h), in which the activities of the biological cycle of living beings are completed. Any dysfunction or severe alteration in this biological clock can lead to a series of diseases, including insomnia, difficulty concentrating and even depression^45^. Enjoying good SQ is important for the physiological regulation process, making sleep restorative, performing the repair and clearance of toxins that are harmful to health^1^ ^,^ ^46^.

It is inferred that shift work, whether fixed or alternated, is a disturbing factor for the biological clock because workers start to have difficulties having regular times to fall asleep and awake, which are important characteristics for a quality sleep period; this fact was proven in the current study when low SQ was found in the workers evaluated, being even more serious in those who worked in alternating shifts. 

In addition to the reality of shift work, extensive working hours can also compromise SQ. The absence of routine sleep schedules means that many professionals are unable to sleep as long as necessary, as some work overtime hours, have two employment contracts and personal and family responsibilities^2^. In this research, the results showed that 28.6% of those who were evaluated had a double employment contract and presented poor SQ. 

A number of studies have shown a strong relationship between SQ and health problems, such as: cognitive decline and attention deficit^47^, increased blood pressure levels and consequent cardiovascular accidents^48^, greater insulin resistance, weight gain^49^, higher incidence of mental ailments such as depression^50^, and neurological diseases such as Alzheimer’s^51^.

Another finding that can be highlighted is the association between SQ and the participants’ work-related characteristics, which was worse in those who had statutory contracts and worked overtime hours. Overtime work increases the weekly working hours, which can result in SQ impairment; the greater the number of hours worked at night, the worse the SQ and mental health states^37^.

The condition of a double employment contract was also considered, which 12 participants (28.6%) claimed to have. Due to the low salaries of Brazilian Nursing workers, it is common to find people who face double or even triple working hours^52^ with a consequent increase in their daily working hours. This fact generates a greater stress condition, which in turn causes activation of the hypothalamic pituitary axis with greater cortisol release, which can promote higher insulin resistance, weight gain caused by hormonal imbalance with increased ghrelin production and reduction of leptin^53^.

It was possible to identify differences in SQ when comparing the type of employment contract, obtaining the worst scores of this quality in those hired by the statutory regime. This fact can be related to the different working hours that such contracts offer, as previously described. With regard to the work shifts, the fixed day professionals presented better SQ results. Among those in the fixed night shift whit a 12-hour work/36-hour off schedule, none presented good SQ. Among those who worked alternating shifts, the results were also worrisome, as most of them presented poor SQ. Regarding the time working in the institution, those who had worked from one to five years obtained the worst SQ results; among those who worked overtime hours, the results of this quality were also worse.

Hospital nurses undergo the work shift changes or 12-hour workdays. Shift change results in difficulty in the chronobiological adaptation, thus impairing the sleep period^54^ and, consequently, its quality.

Another work schedule modality identified among the participants was TNF. This is a modality that favors illness in workers, proven in a study conducted in Portugal, which identified that nurses who work in rotating night shifts were more likely to present sleep problems, fatigue, depression and burnout syndrome, when compared to those who have a regular daytime schedule^55^, thus requiring greater monitoring of their health, aiming to mitigate/avoid physical and mental harms. 

The working time in the institution varied from 6 to 35 years, showing professional stability, results which are consistent with a research study conducted with Nursing professionals from a public hospital in São Paulo^56^. 

WRAs were reported by five nurses (11.9%). Shift work, as well as long workdays or extensive working hours, favor a reduction in SQ, decline in cognitive performance, fatigue and an increase in the risk for burnout^57^, factors that can influence the occurrence of such accidents, in addition to compromising Nursing care quality. Regarding absences from work due to health reasons, 17 (40.5%) confirmed them. Nursing stands out as one of the categories with high risks for stress and illness^58^. It is inferred that the nature of the work performed exposes individuals to varied occupational risks, and exposure to excessive working hours favors illness, requiring workers to be distanced from their activities.

Medication use was high, a fact that seems to be common among Nursing and Physiotherapy professionals, in the case of analgesic drugs^59^. Regarding pain and sleep, sleep impairments trigger and even worsen more intense pain^60^.

In relation to tobacco consumption among the participants, denial of use by the majority is similar to the results of a study carried out with the same professional group that worked in a public hospital in Brazil, in which the sleep parameters and personal characteristics were investigated; however, with regard to the consumption of alcoholic beverages, the results were different, as 54.8% stated that they consumed them^61^. The SQ and life habits results among the participants showed that most of them did not smoke; none of the smokers presented good SQ and among those who consumed alcoholic beverages, the results were slightly worse than those who did not. Consumption of cigarettes and alcoholic beverages can lead to physiological impairments in respiratory, circulatory and metabolic processes, which lead to a worse SQ^62^ ^-^ ^63^. 

There was also high coffee intake. Among those who stated drinking it, the results were worrisome, as only four presented good SQ, 21 had poor quality and 11 already had presence of sleep disorders with an overall mean PSQI value of 9.2. Among the 6 (22.2%) who did not report such consumption, all had poor SQ, with an overall mean PSQI value of 7.5.

Coffee intake among this population is a reality frequently observed in several hospitals; coffee is intended to maintain a state of alert for work, a phenomenon mainly observed when problems arise with sleep or even sleep deprivation, where the workers do not enjoy the necessary period of rest and repair for the body^64^.

The data obtained confirm others from previous studies, which shows that enjoying good SQ is important for nurses^20^ ^,^ ^23^ ^-^ ^24^, who may show more confidence in their professional performance.

The protocols adopted in this study related to sleep investigation, adopted in the Sleep Laboratory of the Ribeirão Preto Medical School of the University of São Paulo, through methodological procedures, may have excluded workers who were possibly presenting the worst cases of impaired health status, as well as it may have made it difficult to find more expressive results. Another limiting aspect referred to the number of participants, which became reduced as a result of the careful use of methodological procedures, which indicates that the results cannot be generalized.

Analyzing nurses’ SQ in relation to their sociodemographic and work-related characteristics, life habits and working conditions may indicate that the evaluation of this physiological process is a possibility for investigating the health of these workers. Such research studies can result in a better QoL and in a reduction of harms to the workers’ health, as well as in the safe care of those assisted by them. 

The meticulous inclusion and exclusion criteria adopted allowed for an important refinement in relation to the results found. *Pittsburgh Sleep Quality Index*(PSQI) was used in its entirety without any type of recategorization, which is closer to the performance of the Index, as envisioned by its authors^31^.

As already explained, this research protocol was composed only by nurses who worked in similar open work units, not generalizing the other realities of the different hospital environments and the nature of work among the professionals. 

It is hoped that, through the findings, it is possible to demonstrate the need for greater monitoring of these workers, particularly those who work in shifts, in order to provide preventive measures, aiming to mitigate the harms to their health and favor them with better QoL and, consequently, with more confidence to develop their work.

## Conclusion

There was impairment in the nurses’ QS; the worst results were among those aged 30-39 years old; and a statistical significance was found in the “living with a partner” variable. There is a need for greater monitoring of the health of these workers, particularly those who work in shifts, in order to provide preventive measures to mitigate the harms to their health. Poor SQ can result in physical and mental illness, decreased work productivity and increased risks for accidents.

It is hoped that, through the findings, it is possible to demonstrate the need for greater monitoring of these workers, particularly those who work in shifts, in order to provide preventive measures, aiming to mitigate the harms to their health and favor them with better QoL and, consequently, with more confidence to develop their work.

Given the situation where SQ is compromised among the participants of this study, it is suggested to carry out further research studies, investigating the Nursing professionals’ sleep, both in the hospital context and in other workplaces, considering the work-related characteristics such as professional category, work sector, wage differences and workloads, in addition to surveys of personal characteristics, such as the evaluation of the these professionals’ chronotype.
